# Effects of caloric restriction and low glycemic index diets associated with metformin on glucose metabolism and cortisol response in overweight/obese subjects: a case series study

**DOI:** 10.1186/s13098-015-0057-9

**Published:** 2015-07-22

**Authors:** Luiz Augusto Casulari, Donatella Dondi, Fabio Celotti, Fábio Vinicius Pires da Silva, Caio Eduardo Gonçalves Reis, Teresa Helena Macedo da Costa

**Affiliations:** Unit of Endocrinology, University Hospital Brasilia, University of Brasilia, Brasilia, Brazil; Dipartimento di Scienze Farmacologiche e Biomolecolari, Sezione di Biomedicina ed Endocrinologia, Università di Milano, Milan, Italy; Postgraduate course in Health Sciences, University of Brasilia, Brasilia, Brazil; Department of Nutrition, School of Health Sciences, University of Brasilia, Brasilia, Brazil; CLINEN – Clínica de Neurologia e Endocrinologia. SCN quadra 1, bloco F, Ed. America Office Tower, sala 1111, Brasília, DF 70711-905 Brazil

**Keywords:** Diabetes, Glucose intolerance, Calorie-restricted diet, Metformin, Cortisol, Receptor

## Abstract

**Background:**

To determine whether cortisol secretion and glucocorticoid receptors in lymphocytes and monocytes are altered in patients with impaired glucose tolerance, and whether treatment with a hypocaloric diet and metformin could interfere with these aspects.

**Methods:**

This is an analytical, interventional, case series study. Patients with impaired glucose tolerance were included. They received 500 mg of metformin twice daily and followed a low glycemic index diet for 16 weeks. Cortisol levels were assessed at 8:00 A.M. before and after use of 0.25 mg of dexamethasone at 11:00 P.M. the day before.

**Results:**

Sixteen subjects (9 men) were included. Normal basal levels of cortisol and adequate responses to the low dose of dexamethasone were observed before and after treatment. There was no significant correlation between the parameters evaluated and cortisol levels. Nevertheless, there was a strong correlation between the number of glucocorticoid receptors, BMI (r = 0.88; *p* = 0.02), and insulin AUC (r = 0.94; *p* = 0.005) before treatment; after treatment, all these associations ceased to exist.

**Conclusion:**

The cortisol secretion remained normal in the group of patients with impaired glucose tolerance. Treatment with metformin and diet did not change this condition. However, glucocorticoid receptor number had a strong correlation with insulin, due to insulin resistance, but this characteristic was lost after treatment.

## Background

Type-2 diabetes mellitus generally affects overweight or obese individuals over 40 years old. These patients have insulin resistance and deficiency [1]. However, before type-2 diabetes mellitus is established, individuals may show the following pre-diabetic conditions: impaired fasting glucose between 100 mg/dl and 125 mg/dl, and impaired glucose tolerance two hours after an oral glucose tolerance test (OGTT), glycemia between 141 mg/dL and 199 mg/dL [1].

Lifestyle changes with adequate nutrition and increased physical activity, with or without treatment with metformin, can prevent or delay the onset of type-2 diabetes mellitus [1–3]. Metformin, which is an insulin action sensitizer, is recommended as a first-choice oral drug for the treatment of type-2 diabetes due to its preventive action in populations at risk [3–5].

In order to potentially prevent the onset of type-2 diabetes in individuals with pre-diabetic symptoms, it is important to study the characteristics involved in the pathophysiology of this condition. A still unsettled problem is the diabetogenic role of cortisol in obese individuals and in patients with metabolic syndrome [6–9].

Several changes in the hypothalamic-pituitary-adrenal (HPA) axis of subjects with abdominal obesity have been described: changes in pulsatile secretion of adrenocorticotropic hormone (ACTH), increased cortisol secretion after laboratory stress tests, hyperresponsivity of the HPA axis to peptides such as the corticotropin-releasing hormone (CRH), associated or not with arginine vasopressin (AVP) (for references, see [10, 11]). There is evidence of peripheral and central alterations of the HPA axis in patients with abdominal obesity [9].

The use of low doses of dexamethasone has been a strategy to detect subtle changes in the negative feedback of cortisol secretion in obese individuals or in those with metabolic syndrome who are not yet diabetic. Alterations in glucose and insulin metabolism can also be identified [10–14]. Nonetheless, results are conflicting. In patients with metabolic syndrome, a higher concentration of cortisol has been observed after treatment with dexamethasone as compared to healthy controls [15]. In obese individuals, cortisol response to dexamethasone was reported as normal [10, 12] or altered [14]. Very few data are available on the effect of insulin resistance on the corticosteroid receptors density [16].

The objectives of this study were to determine whether cortisol secretion is altered in a group of patients with pre-diabetic conditions and whether treatment with a hypocaloric diet and metformin treatment could interfere with this process. In addition, glucocorticoid receptors on mononuclear cells–lymphocytes and monocytes–were analyzed before and after treatment to determine if the receptor expression was affected in this group of pre-diabetic patients.

## Research design and methods

### Study design and ethics

This is an analytical, interventional, case series study designed as a single group clinical trial in pre-diabetic subjects on a hypocaloric low glycemic index diet plus metformin. The study lasted 16 weeks with monthly follow-up visits (total of 5 visits) to verify adhesion to the dietary treatment, to adjust dietary prescription, and to receive medication.

Metformin was kindly donated by Medley Laboratory, Brazil. The dose of 1 g/day metformin was divided into two doses (500 mg) taken at breakfast and dinner. The subjects were instructed to start the metformin treatment with 500 mg per day and after 7 days increase to 500 mg twice a day. In presence of any side effect they were instructed to return to the single dose for 7 days and to increase to twice a day subsequently. During the follow-up period no subjects reported any side effect.

The study protocol was approved by the Ethics Committee in Human Research of the School of Health Sciences at the University of Brasília, Brazil (N. 035/2004). All volunteers were informed of the objectives of the study and signed a written informed consent to participate.

### Subjects

Participants were recruited through public announcements. Inclusion criteria were adults aged between 18 and 50 years old, of both sexes, body mass index from 25 to 35 kg/m². Smokers, pregnant or lactating or post-menopausal women were not included, as well as individuals who had used medication and followed a therapeutic diet in the last two years. Subjects with a fasting blood glucose value higher than 126 mg/dL and/or higher than 200 mg/dL 120 min after the start of the OGTT were also excluded.

The sample size was determined using the software G*Power, version 3.1.9 (Heinrich Heine University Düsseldorf, Düsseldorf, Germany) [17], based on the result from Tam et al. [18] considering the morning cortisol levels as the main variable. A statistical power (1-β) of 80 % and a level of significance (α) of 5 % were considered, which resulted in a sample of 16 subjects, as the necessary number for this study.

All subjects had cortisol inhibition < 1.8 mg/mL at 8:00 A.M. after the ingestion of 2 mg of dexamethasone (Decadron, Merck Sharp & Dohme, São Paulo, Brazil) at 11:00 P.M. of the previous day, thus excluding the occurrence of endogenous Cushing’s syndrome [19].

To evaluate the responsiveness of the hypothalamic-pituitary-adrenal axis to low doses of dexamethasone, a cortisol analysis was conducted after administration of 0.25 mg of dexamethasone at 11:00 P.M. and cortisol levels were measured at 8:00 A.M..

Subjects were also submitted to an oral 75-g glucose tolerance test after an overnight fast. Blood samples were collected from the antecubital vein at 0, 30, 60, 90 and 120 min for insulin and glucose measurements. Individuals with baseline fasting glucose at OGTT <99 mg/dL and >140 mg/dl and glucose levels <200 mg/dL 120 min after the ingestion of oral glucose (impaired glucose tolerance) were selected [1].

Values for the homeostasis model assessment of insulin resistance (HOMA2-IR), HOMA β-cell function (HOMA2- % β), and HOMA insulin sensitivity (HOMA2- % S) were calculated using the computer software HOMA calculator v2.2.2 (University of Oxford; to determine insulin resistance, β-cell function, and insulin sensitivity, respectively [20, 21]. The Cederholm index (CI) was calculated to determine peripheral insulin sensitivity [22]. The calculation, based on an appropriate analytical matrix and unified units, has been utilized in our previous studies in obese subject [23]. Plasma glucose concentration was expressed in mmol/L and insulin concentration in pmol/L. The incremental area under the curve (AUC) for insulin and glucose was calculated excluding any values below the baseline using the trapezoidal method [24].

### Dietary and physical activity assessment

Dietary adherence and physical activity were assessed during the follow-up period according to the methodology described by the Dietary Reference Intakes (DRI) [25].

The subjects followed a hypocaloric low glycemic index diet. Dietary energy reduction was set between 25 and 30 % of the total energy expenditure, and the dietary macronutrient percentages were based on the acceptable macronutrient distribution range [25]. Dietary energy was divided into food groups according to the Brazilian food pyramid. A glycemic index table was created and organized into food groups. At the beginning of the study, participants were instructed in the use of their diet. The table was meant to help participants adhere to the diet.

The adherence to the treatment and the diet was checked in a monthly visit to the laboratory where a follow-up questionnaire was administered and another 30-day card of metformin pills was given to the subjects. They were instructed to bring the used pill card for verification. Adherence to the diet was acknowledged in subjects who made 4–5 visits, did not ingest food that gave them more than the proposed dietary energy and had 4–6 meals per day. The dietary fiber intake was settled to ≥ 70 % of the dietary reference intake of the DRI [25]. Food intake was assessed by repeated 24-h recalls in each visit. To ensure accuracy, a photograph of a food portion guide was shown to all participants estimate the consumed food portions. Each dietary recall was reviewed in the presence of the participant to ensure its accuracy and completeness. Food portions were converted into grams, and total energy intake and consumption of macronutrients and fiber were analyzed using the Nutrition Data System for Research software (version 2011) (NDSR, University of Minnesota, Minneapolis, MN, USA), which includes typical Brazilian foods prepared using standardized recipes. Glycemic index of the diets were calculated to be below 60.

The level of physical activity was assessed using a short-version of the international physical activity questionnaire, and total energy expenditure was estimated by adding the appropriate physical activity level (PAL) to the equations published by the DRI [25].

### Anthropometric and body composition assessment

Body weight was assessed using an electronic platform scale ranging from 0 to 150 kg and precision of 0.1 Kg; height was measured using a scientific stadiometer ranging from 0 to 210 cm and precision of 0.1 cm. BMI was calculated [(body weight (Kg) / height (m^2^)] and classified according to parameters established by the World Health Organization [26]. Waist circumference was measured at the midline between the lowest rib and the iliac crest with precision of 0.1 cm. Body fat percentage was calculated by using a tetrapolar electrical bioimpedance measurement (Bioelectrical Impedance Analysis (BIA) Test–Quantum II RJL^®^ Portable Body Fat Analyzer, USA) [27]. All measurements and classifications were done according to standardized protocols always by the same investigator.

### Biochemical analysis

Fasting state was verified using a glucometer (Roche Diagnostics, Mannheim, Germany) in capillary blood samples. Blood samples drawn in the OGTT or post dexamethasone tests were centrifuged and serum separated. Serum insulin levels were measured by chemiluminescence (Elecsys 2010, *Roche Diagnostics*, Mannheim, Germany), glucose by oxidasis-GOD/POD-automatized (Immulite 2000, DPC, Los Angeles, USA), cortisol by electrochemiluminescence (Elecsys 2010, Roche Diagnostics, Mannheim, Germany), total cholesterol, high density lipoprotein (HDL), and triacylglycerol by enzymatic colorimetric kits (Labtest Diagnostica S.A., Belo Horizonte, Brazil). LDL-cholesterol and VLDL-cholesterol fractions were calculated using the Friedwald equation [28].

### Glucocorticoid receptors

Blood for studying glucocorticoid receptors (GR) was drawn by three Vacutainer ® tubes with EDTA. Blood mononuclear cells (lymphocytes and monocytes) were isolated by the protocol described by Ulmer and Flad [29]. The cells extracted were stored in Eppendorf tubes with a preserving solution and kept in a freezer at -80 °C until sent to the “Dipartimento di Scienze Farmacologiche Biomolecolari, Università di Milano, Sezione di Biomedicina ed Endocrinologia” for analysis. For Western Blotting analysis the cellular lysates were resolved on 7.5 % SDS-polyacrylamide gel electrophoresis (SDS-PAGE), and transferred onto a nitrocellulose membrane. GR protein was visualized with GR rabbit polyclonal antibody (M-20, Santa Cruz Biotechnology, Santa Cruz, Calif., USA), diluted 1:100 [30]. Samples from the last six participants – four women and two men – were analyzed.

### Statistical analysis

A correlation analysis between variables was conducted using Pearson’s correlation coefficient (two-tailed) test. All the characteristics measured, expressed as differences from baseline, were compared after 16 weeks of intervention using the unpaired Wilcoxon test or Student *t* test, according to the absence or presence of normality, respectively, and evaluated by the *Kolmogorov-Smirnov* test. All statistical analyses were done using the Statistical Analysis System software, version 9.1 (SAS Institute Inc., Cary, NC, USA), with statistical significance ≤ 0.05, two-tailed.

## Results

Sixteen subjects were included. Of these, nine were men and seven, women. Their average age was 34.6 SD 7 years (ranging from 19 to 49 years old), but the evaluation of glucocorticoid receptors was conducted in six patients–four men and two women. They were the last six participants included in the study.

Table 1 shows the level of serum cortisol in basal conditions and after suppression by dexamethasone, as well as glucocorticoid receptors in lymphocytes and monocytes. Basal cortisol levels were not altered after treatment (*p* = 0.24). A significant decrease occurred after the use of dexamethasone before (p < 0.001) and after treatment (*p* = 0.01). Cortisol levels after dexamethasone were higher after treatment (*p* = 0.02). Anthropometric and laboratory analysis were not different from that of the whole group. The glucocorticoid receptor expression did not change after diet and metformin use as compared to baseline (*p* = 0.66).

**Table 1:** Cortisol levels in basal conditions and after suppression by dexamethasone; glucocorticoid receptors in lymphocytes and monocytes at baseline and 16 weeks after a hypocaloric low glycemic index diet and metformin use in 16 subjects

Variables	Baseline		16 weeks		Δ	*p* ^a^
	Mean	SD	Mean	SD		
Basal cortisol (μg/dL)	7.19 ^b^	3.35	8.69 ^c^	4.55	1.50	0.24
Post-dexamethasone cortisol (μg/dL)	4.27 ^b^	2.85	5.99 ^c^	5.31	1.72	0.02
Glucocorticoid receptors ^d^	23,390	8052	26,650	9408	3260	0.66

Data are expressed as mean and standard deviation (SD)

^a^16 weeks *vs.* basal = paired Student *t* test

^b^p < 0.001 basal cortisol *vs*. post-dexamethasone cortisol = paired Student *t* test

^c^ *p* = 0.01 basal cortisol *vs*. post-dexamethasone cortisol = Wilcoxon test

^d^ *n* = 6 subjects (4 male, 2 female)

Table 2 shows the correlation coefficient of selected variables and basal cortisol level, post-dexamethasone cortisol level and number of glucocorticoid receptors in lymphocytes and monocytes in basal conditions and after 16 weeks of intervention. A positive correlation was observed between BMI and basal cortisol (r = 0.50; *p* = 0.05) after treatment. Glucocorticoid receptors levels before treatment were strongly and positively correlated with BMI (r + 0.88; *p* = 0.02) and insulin AUC (r +0.94; *p* = 0.005). After intervention, there were no correlations with BMI (r -0.19; *p* = 0.71) and the correlation with insulin AUC became negative and non-statistically significant (r -0.72; *p* = 0.27). There were significant negative correlations between cortisol responses to dexamethasone and triglycerides (r - 0.68; *p* = 0.05) and VDLc (r – 0.67; *p* = 0.05). These correlations were also found after treatment (r - 0.56; *p* = 0.04 and r - 0.55; *p* = 0.04). A relevant positive correlation of insulin (r + 0.76; *p* = 0.08), HOMA-IR (r + 0.78; *p* = 0.07), HOMA2- % β (r + 0.75; *p* = 0.09) and a negative correlation of HOMA2- % S (r - 0.75; *p* = 0.07) was observed with the glucocorticoid receptors in basal conditions, even if the values did not reach the level of statistical significance.

**Table 2:** Pearson correlation of selected variables and basal cortisol level, post-dexamethasone cortisol level and number of glucocorticoid receptors in lymphocytes and monocytes in basal conditions and 16 weeks after a hypocaloric low glycemic index diet and metformin use in 16 subjects

Variables	Basal cortisol				Post-dexamethasone cortisol				Glucocorticoid receptors^b^			
	Basal		16 weeks		Basal		16 weeks		Basal		16 weeks	
	r	*p*	r	*p*	r	*p*	r	*p*	r	*p*	r	*p*
BMI ^a^	-0.44	0.09	0.50	0.05	0.28	0.80	0.35	0.22	0.88	0.02	-0.19	0.71
Glucose	0.39	0.14	-0.22	0.42	-0.33	0.38	-0.02	0.94	-0.59	0.22	-0.48	0.33
AUC glucose^a^	-0.04	0.87	-0.19	0.49	-0.49	0.11	-0.49	0.07	-0.50	0.31	0.53	0.27
Insulin	-0.21	0.43	-0.24	0.37	0.54	0.91	-0.11	0.71	0.76	0.08	-0.44	0.38
AUC insulin^a^	-0.20	0.45	-0.31	0.24	0.08	0.89	-0.21	0.46	0.94	0.005	-0.72	0.27
Cederholm index	0.06	0.83	0.11	0.68	0.15	0.74	0.38	0.18	-0.53	0.28	0.38	0.46
HOMA2-IR^a^	-0.21	0.45	0.06	0.83	0.50	0.96	0.27	0.37	0.78	0.07	-0.58	0.23
HOMA2-%β^a^	-0.38	0.16	0.17	0.54	0.56	0.57	0.09	0.76	0.75	0.09	-0.45	0.37
HOMA2-%S^a^	0.01	0.97	-0.25	0.37	-0.40	0.50	-0.47	0.10	-0.75	0.07	0.75	0.09
Triglyceride	-0.10	0.72	-0.37	0.16	-0.68	0.05	-0.56	0.04	0.05	0.92	0.16	0.76
VLDL-c^a^	-0.09	0.73	-0.36	0.17	-0.67	0.05	-0.55	0.04	0.03	0.95	0.17	0.74

^a^Abbreviations: BMI = body mass index; AUC glucose = *area under the glycaemic curve;* AUC insulin = a*rea under the insulinaemic curve;* HOMA2-%β = homeostasis model assessment β-cell function; HOMA2-IR = homeostasis model assessment insulin resistance; HOMA2-%S = homeostasis model assessment insulin sensitivity; VLDL-c = very low density lipoprotein cholesterol

^b^6 subjects (4 male and 2 female)

There were no significant correlations among cortisol and glucocorticoid receptor levels with body weight, waist circumference, body fat, total cholesterol, HDLc, and LDLc (data not shown).

## Discussion

The epidemic rise of obesity and type-2 diabetes worldwide is partly due to the excessive intake of inadequate nutrients and little physical activity [2]. Before the onset of type-2 diabetes, predisposed individuals develop pre-diabetic conditions that lead to the disease. The progression to diabetes is weak for subjects with impaired fasting glucose, intermediate for those with impaired glucose tolerance and strong for those with both conditions [2].

In this study, overweight or obese individuals with impaired glucose tolerance were treated with metformin and a hypocaloric low glycemic index diet for four months. The clinical results of the therapeutical association of the diet and metformin in these patients were previously published. The treatment produced a significant decrease in body weight, BMI, waist circumference, and body fat over the follow-up period. No change in physical activity was observed during the intervention weeks. In addition, a significant reduction in CI, HOMA2- % β, triglycerides, and VLDL levels was observed [31].

The homeostatic model assessment (HOMA) [20] used to quantify insulin resistance and beta-cell function indicates that the treatment has produced a reduction of the beta-cell function (HOMA2- % β), which was no longer required to secrete a greater amount of insulin since insulin sensitivity was increased, as shown by a higher Cederholm Index (CI) [22].

Cortisol secretion in these pre-diabetic subjects was tested evaluating plasma cortisol and utilizing the low doses of dexamethasone suppression test in an attempt to detect subtle changes in the feedback of cortisol control in the hypothalamus and pituitary gland. Cortisol levels and physiological cortisol suppression occurred in response to low doses of dexamethasone, before and after intervention, in agreement with previous investigations conducted to verify normal and abnormal secretion of this hormone [9, 10, 19]. We can infer from these results that cortisol secretion is preserved in obese individuals in pre-diabetic condition.

The HPA axis activity in obese and type 1 diabetic patients has been investigated in several studies with conflicting results. In obese individuals cortisol levels have been reported to be normal [32, 33], while in other studies fasting and postprandial cortisol secretion in obese patients has been described as lower than in lean subjects [34]. In agreement with the findings of this study several authors [10, 12, 14] have reported normal cortisol suppression tests in response to low doses dexamethasone in obese individuals.

In patients with type 2 diabetes the hypothalamic pituitary-adrenal (HPA) axis activity appears increased in most studies: elevation of ACTH [35, 36], of basal [35, 37, 38] and suppressed serum cortisol (after dexamethasone test) [39, 40] and of late-night salivary cortisol levels [41] have been reported. In particular the presence of chronic complications of type 2 diabetes (i.e., macroangiopathy, retinopathy, and neuropathy) have been associated to with HPA axis overactivity [37, 42–47].

The corticosteroid receptor density measured in a subset of our patients in leukocytes does not show a significant variation between the pre and post-treatment conditions. To the authors’ knowledge a single paper is available in the literature on the relationships between insulin secretion, insulin resistance and corticosteroid receptors. In agreement with the results of the study here presented, Islam et al. [16] reported, in a study including 78 normo or slightly hyperglycemic subjects, that glucocorticoid receptor concentration in leukocytes is significantly and positively correlated with insulin resistance and BMI.

However, it is worth of note that there is a strong correlation between corticosteroid receptors and insulin AUC, BMI and, to a minor extent, insulin levels and insulin resistance in the pre-treatment period, when the insulin secretion was increased. After metformin and hypocaloric diet treatment, when insulin sensitivity improved, this correlation disappears. The beneficial effect of this type of metformin and hypocaloric diet treatment in improving insulin sensitivity was also described in two previous articles by some of the authors of our research group. In the first one, quoted above, metformin and hypocaloric diet showed beneficial effects in anthropometric and metabolic parameters of the same group of subjects with impaired glucose tolerance [31]. In the second study, an increase in the levels of total and free testosterone was observed in subjects with hypogonadotropic hypogonadism with metabolic syndrome [48].

The mechanisms involved in metformin hypoglycemic action in diabetic or glucose intolerant subjects are still unknown [49–51]. Metformin has a glucose lowering effect related to reduction in hepatic gluconeogenesis. The action is partially related to antagonism to glucagon activity [49], but also to stimulation of 5’-AMP-activated protein kinase, which confers insulin sensitivity, mainly by modulating lipid metabolism [49–51]. Recently, it has been shown that oral administration of 1000 mg of metformin causes cortisol metabolite fluctuation, indicating that the neuroendocrine system may be involved in metformin’s antidiabetic effect [51]. There is in vitro and in vivo evidence that the antidiabetic effects of metformin are related to the gluconeogenetc actions of cortisol action. It has been proposed that the mechanism involved is a reduction of POMC/adrenocorticotropic hormone/cortisol levels following AMPK/liver X receptor α phosphorylation in the pituitary [51].

## Conclusion

Cortisol secretion was not altered in this group of patients with impaired glucose tolerance, and treatment did not change this condition. However, glucocorticoid receptors are strongly correlated with insulin secretion and lose this characteristic after treatment.
